# Manipulating the soil microbiomes during a community recovery process with plant beneficial species for the suppression of *Fusarium* wilt of watermelon

**DOI:** 10.1186/s13568-021-01225-5

**Published:** 2021-06-12

**Authors:** Xu Zhang, Chao Xue, Dan Fang, Xiaohui He, Mengyu Wei, Chenjin Zhuo, Junyao Jin, Biao Shen, Rong Li, Ning Ling, Qirong Shen

**Affiliations:** Jiangsu Provincial Key Lab for Solid Organic Waste Utilization, National Engineering Research Center for Organic-Based Fertilizers, Jiangsu Collaborative Innovation Center for Solid Organic Waste Resource Utilization, Nanjing Agricultural University, Nanjing, 210095 China

**Keywords:** Watermelon *Fusarium* wilt disease, Soil microbial resistance and resilience, Dazomet fumigation, Biological organic fertilizer, Soil microbial assembly

## Abstract

*Fusarium* wilt is a devastating disease which impacts watermelon production. Soil fumigation using dazomet followed by biological organic fertilizer was applied to suppress the *Fusarium* wilt disease. We propose that fumigation suppresses the soil indigenous community, especially the soil-borne pathogens, while the utilization of bio-organic fertilizer facilitates the recovery of the soil microbiome to a beneficial, suppressive state through the introduction of plant growth-promoting microorganisms. Greenhouse experiment showed that applied biological organic fertilizer after dazomet fumigation effective restrain the disease incidence with a 93.6% disease control. Fumigation strongly decreased soil microbial diversity and altered relative taxa abundances, suggesting the possibility of niche release by the resident soil microbial community. Fumigation followed by bio-fertilizer transformed the soil microbial community composition and resulted in higher relative abundances of beneficial microbial groups such as *Bacillus* (8.5%) and *Trichoderma* (13.5%), coupled with lower *Fusarium* abundance compared to other treatments. Network analysis illustrated that soil fumigation decreased interactions within the soil microbial community with less nodes and links while bio-fertilizer addition promoted node interactions. In addition, bio-fertilizer addition after fumigation resulted in the beneficial species becoming the key network connectors. Collectively, fumigation appears to release the resident soil niche resulting in lower diversity while the beneficial microbes introduced by bio-fertilizer addition colonize these niches, leading to a more complex community with fewer pathogens that suppresses *Fusarium* wilt disease incidence.

**Supplementary Information:**

The online version contains supplementary material available at 10.1186/s13568-021-01225-5.

## Introduction

Watermelon *Fusarium* wilt as a serious plant disease worldwide was arose from the pathogenic fungus *Fusarium oxysporum* f. sp. *niveum* (FON) (Ling et al. 2010a). which seriously restrains watermelon production worldwide (Zhou and Everts 2004). Dazomet (tetrahydro-3,5-dimethyl-2H-1,3,5-thiadiazine-2-thione) is often employed to suppress serious *Fusarium* infection in watermelon (Slusarski and Pietr 2009; Tian et al. 2014). When dazomet is applied to moist and hyperthermal soils, the fumigant decomposes into methyl isothiocyanate (MITC), which can effectively suppress fungi, nematodes, and weeds (Saeed et al. 2007). Many beneficial antergic species, for instance, *Bacillus* spp., and *Trichoderma* spp., which isolated from suppressive soil were utilized for the suppression of *Fusarium* wilt disease already (La Fuente et al. 2006; Qiu et al. 2012; Yuan et al. 2016). Beneficial species premixed with compost and amino acid as effective fertilizers in the suppression of many cash crops soil-borne disease have been widely reported (Luo et al. 2010; Qiu et al. 2012; Wu et al. 2014). The extra spaces and nutrients provided by bio-organic amendments for the antergic species that facilitates their colonization and pathogen antagonism (Xiong et al. 2017a). Such fertilizers increase the soil fertility and reduce the adverse impact of disease pathogens, thereby improve the plant vitality (Bailey and Lazarovits 2003). Nevertheless, few is understood concerning the underlying mechanisms of fumigation using dazomet followed by biological organic fertilizer amendment during the process of soil-borne disease inhibition.

Soil microbes have a determinate place within the sustainability of soil biological activity, maintaining ecological balance for the soil ecosystem and plant production through their metabolic activities and energy exchanges (Zarraonaindia et al. 2015). It is necessary to understand the contribution of soil microbial community resistance and resilience during the manipulation of the resident soil microflora for the disease suppression (Allison and Martiny 2008). A outside disturbance is often caused by biotic or abiotic elements, which leads to a microbial community tiny destabilization or a ‘stress’, such as the reaction from the independent or the whole ecological system. On the basis of their length of time, disturbances are usually divided into pulses and presses, which affect the soil physicochemical properties (Rykiel 1985). In general, fumigation can be recognized as a ‘disturbance’, which is a pulse disturbance as it is a short-term event. Dazomet fumigation may exert a toxic impact on the microbial community due to the methyl-isothiocyanate and sulphuric acid it volatilizes which negatively effects soil microorganisms, including the beneficial species (Scopa and Dumontet 2007). Ultimately, the recovery of soil productivity is dependent on the re-establishment of the soil microbial activity and the creation of a biological environment for soil diseases control (Eo and Park 2014). In consideration of the decisive importance of maintaining soil microbial ecosystems for long-term productivity, large numbers of vigor on realizing the reactivity (including resistance and resilience) of microbial community to extraneous disturbances has been put into effect (Wertz et al. 2007; Fujino et al. 2008). Fumigation followed by the biological organic fertilizer application amendment is able to suppress soil-borne wilt disease through decreasing the number of pathogens and by modifying the resident soil microbial community diversity and/or structure.

Our hypothesis is that soil fumigation will overcome the inherent resistance of microbial community also suppress the resident soil community, including both pathogens and beneficial species. Beneficial microbiota introduced through bio-fertilizer application will further suppress pathogen growth while promoting crop viability through manipulating the resident microbial community during the period of community resilience. To test our hypothesis, greenhouse experiment was carried out to confirm the effectiveness of the biological organic fertilizer application after dazomet fumigation on the control of watermelon *Fusarium* wilt disease. The soil samples were gathered, and soil microbial composition and diversity were accessed after soil fumigation and biofertilizer application. Microbial ecological network was established with the purpose of exploring community interaction and complexity to reveal the effect of an integrated agricultural strategy on soil microflora.

## Materials and methods

### Microorganisms, organic and biological organic fertilizer preparation

Antagonistic bacterium *Bacillus amyloliquefaciens* SQR9 (CGMCC NO.5808, China Microbial Culture Collection Committee General Microbiology Center) and the fungal strain *Trichoderma guizhouense* NJAU4742 (CGMCC NO.12166, China Microbial Culture Collection Committee General Microbiology Center) were separated by the Jiangsu Provincial Key Lab for Organic Solid Waste Utilization, Nanjing Agricultural University. Both strains showed negative impact on the fungal pathogen *F. oxysporum* growth (Cao et al. 2011; Yuan et al. 2016). Bio-fertilizer preparation process was described previously, at a word, a certain number of antagonistic strain SQR9 and NJAU4742 were added into compost for second solid fermentation and finally reach the concentration of 10^9^ CFU g^−1^ and 10^8^ CFU g^−1^, respectively (Ling et al. 2010b; Zhang et al. 2008).

### Field experiment

Field experiment was conducted in Huaian (33°35′ N, 119°01′ E), Jiangsu Province, China from March to July 2017. The average temperature in the greenhouse was 25 °C during the whole process of watermelon growth. Watermelon was continuously cultivated in the greenhouse for more than 4 years (eight growing seasons). The *Fusarium* wilt disease incidence reached up to 80% with approximately 10^5^ CFU of FON per gram soil in the previous growing season. The soil has a pH value of 6.15, a total nitrogen content of 0.62 g kg^−1^ and available phosphorus, potassium contents of 60, 256 mg kg^−1^ respectively.

Two treatments (FOF, FBOF) and a control (OF) were set to test the effect of dazomet fumigation followed by biological organic fertilizer on watermelon *Fusarium* wilt disease: (I) Soil fumigated with dazomet (375 kg per ha) and treated with biological organic fertilizer (7500 kg per ha) was assigned as FBOF; (II) Soil treated with organic fertilizer (7500 kg per ha) following fumigation with dazomet (375 kg per ha) was assigned as FOF. (III) Soil treated with organic fertilizer (7500 kg per ha) without fumigation served as control (OF). Bio-organic fertilizer is a mixture of organic fertilizer and agricultural amino acid inoculated with *Bacillus amyloliquefaciens* SQR9 and *Trichoderma guizhouense* NJAU4742 which were separated by our laboratory to suppress soil-borne diseases. Nutrients in the control and two treatments were equal.

The dazomet was applied into the soil evenly after soil was tilled completely. Then the soil was covered by plastic film after watered to achieve 40% moisture. The plastic film was took away after 10 days, which meant fumigation was terminated. Fertilizers were added to the soil after another 7 days followed by another tillage. Watermelon seedlings (Sumeng No.6) which grown to two tender buds were transplanted into the soil. Eight replicates (5 m^2^ for each) with 10 seedlings were set in each treatment. Watermelon seeds were first soaked in warm water at 60 °C, stirred to room temperature, sterilized with 5% sodium hypochlorite for 3 min, then germinated in incubator after washed with sterile water for 3–4 times, and transplanted after they grow to two leaves.

After harvested, plants which exerted *Fusarium* wilt disease symptoms were counted, then soil samples were collected. In this study, the percentage of diseased plants to the total plants was computed to express the morbidity.

### Soil sampling and total DNA extraction

Soil from the top 15 cm was sampled used five-point sampling method after watermelons were harvested. Briefly, soil from five different holes were collected and mixed as one soil sample of a plot. Soil samples collected from each treatment were placed into ice box for retaining freshness then transported to the laboratory. Finally, soil total DNA was extracted under the manufacturer’s protocol of the Power soil DNA Extraction kit (MOBIO Laboratories, 108 Carlsbad, CA, USA).

### Quantification of pathogens

*Fusarium oxysporum* selective medium was used for plate counting which one liter containing 1 g K_2_HPO_4_, 0.5 g KCl, 0.5 g MgSO_4_, 0.01 g Fe-Na-EDTA, 2 g l-asparagin, 20 g d-galactose and 1000 mL water (Komada 1975). 5 g of soil in each sample was added to a 150 mL conical flask containing 45 mL of sterilized distilled water. Next, the soil suspension was diluted and applied on the medium after vibrating for 30 min at 170 rpm under room temperature on the shaker.

### Soil total DNA amplification and sequencing

Primer set 338F (5′-ACTCCTACGGGAGGCAGCAG-3′) and 806R (5′-GGACTACHVGGGTWTCTAAT-3′) (Xu et al. 2016) were utilized for the amplification of the 16S rRNA V3-V4 variable regions. ITS3F (5′-GCATCGATGAAGAACGCAGC-3′) and ITS4R (5′- TCCTCCGCTTATTGATATGC-3′) (White et al. 1990) were utilized for the amplification of the fungal internal transcribed spacer (ITS). All the amplifications were conducted in a 50 μL mixture including 2 μL DNA template, 10 μL 5 × Q5 reaction buffer, 4 μL of 2.5 mM dNTPs, 2.5 μL of each primer and 0.5 μL Q5 high-fidelity DNA polymerase (New England Biolabs, UK) using Eppendorf Mastercycler nexus gradient (Eppendorf AG, Hamburg, Gemany). The conditions of Polymerase Chain Reaction (PCR) were 98 °C for 2 min, and then 35 cycles of 10 s at 98 °C, 15 s under 59 °C, 30 s at 72 °C, 72 °C for 2 min, followed by storage at 10 °C.

### Bioinformatics analysis

Sequence analyses were mainly accomplished with QIIME (Quantitative Insights Into Microbial Ecology) pipeline (Caporaso et al. 2010). Briefly, raw paired-end sequences were assembled, and then low quality (q > 0.5) and short reads (bp < 200) were filtered. The assembled OTUs were clustered at 3% dissimilarity and OTU tables were performed. Alpha diversity concluding Chao and Shannon indices were calculated under the OTU table. Non-Metric Multidimensional Scaling (NMDS) analysis based on Bray–Curtis distances was figured up using vegan packages in R (version 3.3.0). Manhattan plots were carried out to explore the differences between the control and treatments at the phyla level using edgeR and dplyr packages in R (version 3.3.0). Raw sequences from each sample were uploaded to the NCBI Sequence Read Archive (SRA) database (Accession Number: SUB4843217).

### Network analysis

Phylogenetic molecular ecological networks (pMENs) of different treatments were performed on the basis of the Molecular Ecological Network Analyses Pipeline (MENAP) (http://129.15.40.240/mena/) (Deng et al. 2012; Zhou et al. 2010). First, a matrix of OTU-based microbial variables table file was formed as the formats required. Then, a pairwise Pearson correlation between any two OTUs was calculated by an RMT-based measure based on the abundance data. The network characteristics were determined by module detection calculations. Finally, the network graph was visualized by Gephi platform (Ling et al. 2016; Zhou et al. 2010, 2011). Zi and Pi values were calculated to represent the topological roles of each nodes and the threshold of them for classifying these OTUs were 2.5 and 0.62, respectively.

### Statistical analysis

Multiple comparisons between samples were carried out utilizing a one-way ANOVA (Duncan test) with significance indicated at *P* < 0.05 using IBM SPSS 19.0 (IBM Corporation, New York, United States). Mantel tests and pearson correlation analysis between microbial relative abundances and morbidity were performed using R (version 3.3.0).

## Results

### Disease incidence and quantification of pathogens

**Fig. 1:**
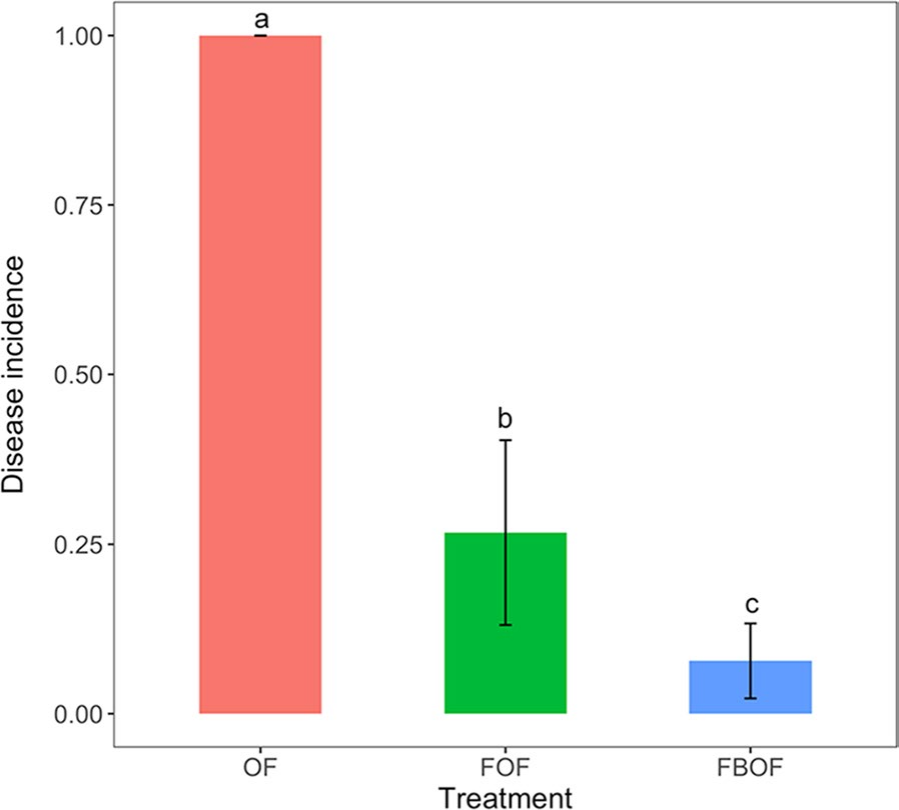
Disease incidence of watermelon *Fusarium* wilt in different treatments. *OF* organic fertilizer, *FOF* dazomet fumigation followed by organic fertilizer, *FBOF* dazomet fumigation followed by biological organic fertilizer. Different lowercases indicate a significant difference at the 0.05 probability level according to the Duncan test

The watermelon *Fusarium* wilt incidence in control (OF) treatment reached up to 100% while a lower incidence rate (29.5%) of the fumigation (FOF) treatment was observed. Compared with the control treatment, the fumigation followed by biological organic fertilizer (FBOF) treatment significantly decreased the disease incidence to 6.4% (*P* < 0.05) (Fig. 1) (93.6% disease control).

Compared to OF treatment, the population of pathogen decreased significantly in fumigation treatments (FOF and FBOF), pathogens were almost undetectable in these two treatments (Additional file 1: Figure S1).

### Microbial community diversity

The soil microbial communities (including bacteria and fungi) alpha-diversity (Chao and Shannon) indices in the fumigation (FOF and FBOF) treatments were significantly lower compared with OF treatment. However, there was no significant difference between FOF and FBOF treaments (Table 1).

**Table 1:** Soil microbial diversity indices

	Treatment	Chao	Shannon
Fungi	OF	2188 ± 230a	6.14 ± 0.29a
	FOF	1790 ± 283b	5.14 ± 1.04b
	FBOF	1782 ± 461b	4.59 ± 0.59b
Bacteria	OF	6730 ± 683a	11.15 ± 0.24a
	FOF	5227 ± 1135b	10.44 ± 0.74b
	FBOF	5572 ± 891b	10.30 ± 0.58b

Values are means ± standard deviation (n = 8). Different lowercases indicate a significant difference among all treatments (*P* < 0.05; Duncan test)

*OF* organic fertilizer, *FOF* dazomet fumigation coupled with organic fertilizer, *FBOF* dazomet fumigation coupled with biological organic fertilizer

**Fig. 2:**
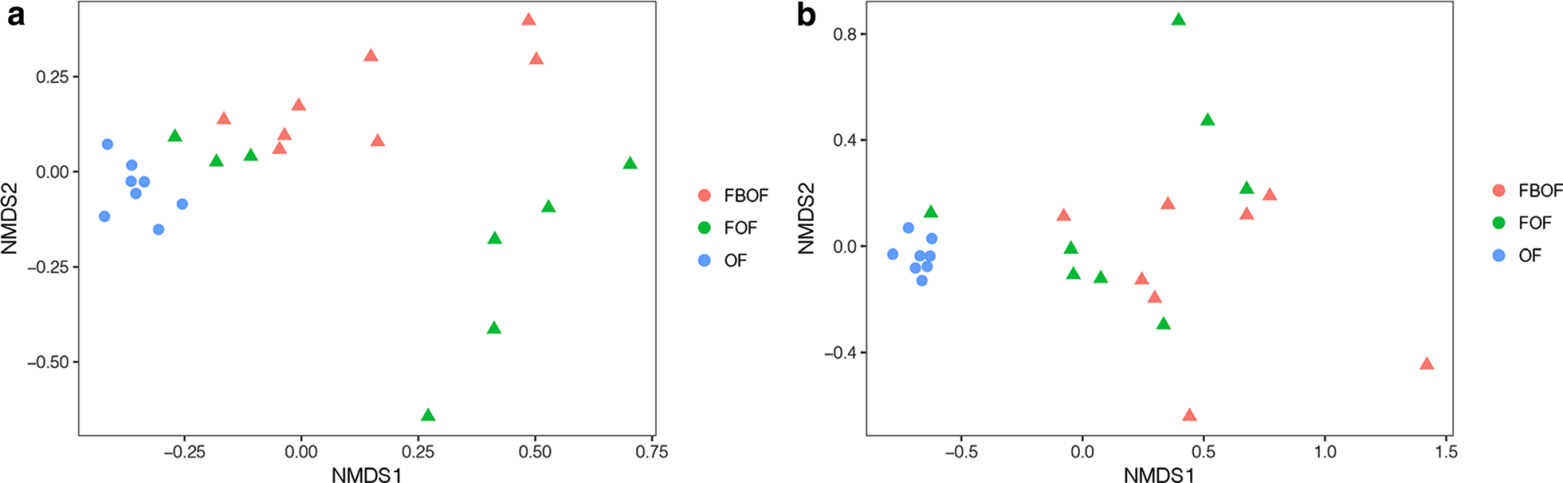
Nonmetric Multidimensional scaling (NMDS) analysis in bacterial (**a**) and fungal (**b**) microbiome between all sample sets. *OF* organic fertilizer, *FOF* dazomet fumigation followed by organic fertilizer, *FBOF* dazomet fumigation followed by biological organic fertilizer

Nonmetric multidimensional scale (NMDS) analysis based on the Bray–Curtis distance illustrated significant differences in soil microbial community composition between the three treatments (PERMANOVA, *P* < 0.05) (Fig. 2). Compared to control treatment, the microbial communities in FOF and FBOF treatments were relatively similar.

### Microbial community composition

Under the bacterial phylum level, *Proteobacteria* were the most abundant, comprising 31.3%, 35.3%, and 35.0% of the bacterial population in the OF, FOF, and FBOF treated soils, respectively (Additional file 1: Figure S2). As observed, the relative abundance of *Firmicutes* (8.5%), *TM7* (5.7%), *Gemmatimonadetes* (5.4%), and *Actinobacteria* (5.3%) in FBOF treatment were significantly higher than OF and FOF treatments. Conversely, the relative abundance of *Acidobacteria* in FBOF treatment (2.6%) dramatic lower than that in OF (7.9%) and FOF (4.1%) treatments.

**Fig. 3:**
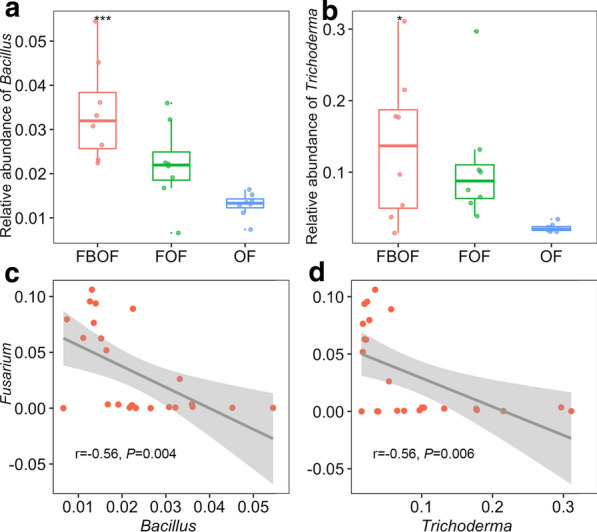
The relative abundance (RA) of genus *Bacillus* (**a**) and *Trichoderma* (**b**) in the three treatments and Pearson correlations (r) between RA of *Bacillus* (**c**) and *Trichoderma* (**d**) with RA of *Fusarium*. The asterisk indicates significant differences among the treatments, as defined by Duncan test (*P* < 0.05). *OF* organic fertilizer, *FOF* dazomet fumigation followed by organic fertilizer, *FBOF* dazomet fumigation followed by biological organic fertilizer

The relative abundance of the fungi *Arthrobotrys* (16.5%) and *Phialemonium* (8.1%) in FBOF treatment were significantly higher compared to OF and FOF treatments (Additional file 1: Figure S2). The genus *Trichoderma* accounted for 2.2%, 10.8% and 13.5% of the fungal community in the OF, FOF and FBOF treatments, respectively. The relative abundance of *Fusarium* in OF (7.9%) was the highest among the three treatments. Compared to OF, the FBOF treatment significantly increased the abundance of *Firmicutes* (Additional file 1: Figure S3), *Bacillus*, and *Trichoderma* (Fig. 3).

A Mantel test indicated that the bacterial (ρ = 0.30, *P* < 0.01) and fungal (ρ = 0.52, *P* < 0.01) community compositions were significantly correlated with disease incidence (Additional file 1: Table S1). Among the dominant microbial genera (relative abundance > 1%), *Bacillus* (r = − 0.56, *P* = 0.004) and *Trichoderma* (r = − 0.56, *P* = 0.006) illustrated significant negative correlations with *Fusarium* relative abundance (Fig. 3).

### Different effects of amendments on soil microbial molecular ecological network

**Fig. 4:**
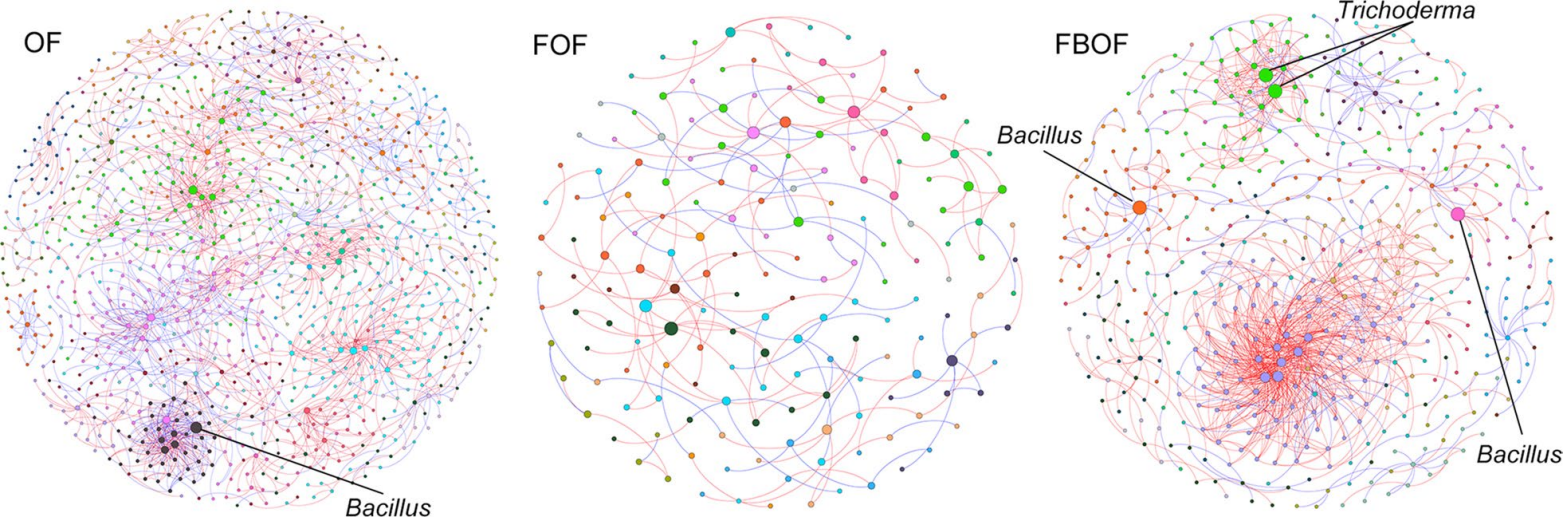
Different networks performed by OTU tables based on RMT analysis. *OF* organic fertilizer, *FOF* dazomet fumigation followed by organic fertilizer, *FBOF* dazomet fumigation followed by biological organic fertilizer. Different networks represent random matrix theory co-occurrence models for each treatment. Different nodes mean different OTUs, and links between the nodes indicate significant correlation. Modules are randomly colored for, and modules with more than 5 nodes were shown

Network plots revealed varying microbial co-occurrence network structures among the three treatments (Fig. 4). Different R^2^ values of 0.835, 0.951 and 0.918 for FBOF, FOF and OF treatments, respectively, indicating networks formed possess scale-free properties (Additional file 1: Table. S2). With more links in FBOF treatment than FOF treatment, the biological organic fertilizer application after fumigation increased the connectivity, resulting in a more complex soil microbial community.

The topological character of each nodes was demonstrated with Zi-Pi plot (Additional file 1: Figure S4). Majority (96.4%) of the nodes were peripherals as most of their links inside their modules. About 3.5% of the nodes were module hubs while only one Bacteroidetes OTU was a connector. Among all the module hubs, a total of 43, 6, and 24 OTUs belonged to the OF, FOF and FBOF treatments, respectively. Three of the 24 FBOF module hubs were *Firmicutes*, and two of them were closely related to *Bacillus*. Three module hubs in FBOF were assigned to *Ascomycota,* of which two were closely related to *Trichoderma*. The other hubs belonged to varying taxa (i.e., *Proteobacteria*, *Gemmatimonadetes*, *Chloroflexi*, and *Acidobacteria*) (Additional file 1: Table S3).

## Discussion

As we aimed to explore the underlying mechanisms that link dazomet fumigation followed by biological organic fertilizer application with watermelon *Fusarium* wilt disease suppression. Two fumigation treatments, especially FBOF (applied biological organic fertilizer after fumigation), were effective in reducing Fusarium wilt disease incidence. This demonstrated that dazomet fumigation was highly effective in suppressing *Fusarium* wilt disease, as supported by previous studies (Tian et al. 2014). In the meantime, treatment with only organic fertilizer (OF) resulted in the highest disease incidence, demonstrating that organic fertilizer application alone was not effective in inducing disease suppression in severe diseased area. A previous study indicated that the application of organic fertilizer alone was ineffective and possibly propitious to the growth of pathogens (Bonanomi et al. 2010). Thus, the utilization of dazomet followed by a biological organic fertilizer amendment was a better alternative for controlling *Fusarium* wilt disease.

In our greenhouse study, we observed that the microbial communities were principally sensitive to dazomet fumigation and resilient to the disturbance caused by the combination of dazomet fumigation and bio-fertilizer application. In comparison to organic fertilizer amendment alone (OF), both the Chao and Shannon indices exhibited dramatic decreases in microbial richness and diversity in the fumigated treatments. The result is identical with previous findings that fumigation could reduce microbial biodiversity (Fu et al. 2012; Griffiths et al. 2000). The control treatment exhibited significantly higher richness and abundance than the dazomet-treated samples further supporting the impact of fumigation, as dazomet is widely known to suppress the growing of microbial communities (Eo and Park 2014; Scopa and Dumontet 2007). On the basis of the insurance hypothesis, biodiversity loss might lead to a reduction in ecosystem stability (Griffiths and Philippot 2013). Previous experiments have supported the hypothesis by using differential gradient fumigation to decrease soil biodiversity, and the lower biodiversity in soil indicated a reduced resilience in plant decomposition after disturbance (Griffiths et al. 2000).

It seems that the compositional transformation of the intrinsic *Fusarium*-dominated soil microbial community to a re-assembled non-disease community was the basis for disease suppression (van Elsas et al. 2012). Supporting this speculation, we observed the bacterial and fungal community composition of OF treatment was distinctly different from the other two treatments (FOF, FBOF) which supports the differences in the alpha-diversities between the treatments. This confirms previous results where fumigation often led to diversification in community composition (Ibekwe et al. 2001; Omirou et al. 2010; Shen et al. 2018). In addition, the initial disturbance by fumigation was able to break the stability of soil microbial community by decreasing community diversity and alter the community structure and potentially releasing the available niche space, temporarily decreasing species competition. Then the invader whether colonized in the soil depends on its ability to achieve the available niche space within the disturbed community (Kinnunen et al. 2016).

Two types of fertilizer in our greenhouse study are composts rich C, N, and exogenous microbes (*Bacillus* and *Trichoderma* in bio-fertilizer). When applied to soil, they advance the soil fertility, ameliorate soil structure and increase or decrease several microbial abundances and activities (Ling et al. 2014; Suleiman et al. 2016). Hence, different amendments endowed distinct microbial communities. Lower abundance of the pathogen *Fusarium* and higher abundances of the antagonistic agents *Bacillus* and *Trichoderma* introduced by biological organic fertilizer are likely related to *Fusarium* wilt disease incidence in this study. Mantel tests revealed that microbial community composition might be a key factor in disease suppression. The highest relative abundance of *Firmicutes* identified within the FBOF treatment revealed that they may be involved in disease suppression in these soils (Trivedi et al. 2017; Xiong et al. 2017b). The *Firmicutes* include numerous potential biocontrol agents and has been reported with a higher abundance in suppressive soils of different soil-borne disease systems (Rosenzweig et al. 2012; Shen et al. 2015). At the genus level, *Bacillus*, which was widely used to suppress soil-borne wilt disease, were significant higher in FBOF than other two treatments (Cao et al. 2011; Zhang et al. 2008). Due to their broad-spectrum antibiotic activity and the ability of form endospores, the *Bacillus* species possess several advantages in excess of other agents for protection against pathogens (Cavaglieri et al. 2005). Previous research on cucurbits showed that fumigation can kill the mycelia of *F. oxysporum* of cucumber and then result in a dramatic abundance diminution of the pathogen (Li et al. 2016). For further impact restrained the relative abundance of *Fusarium*, biological organic fertilizer application builds on the initial express of fumigation, which due to the disease suppressive capacity of microbial populations contained within the biological organic fertilizer. It should be noticed that pathogenic or non-pathogenic *Fusarium* were not separately quantified in this study yet. Nevertheless, field disease incidence revealed that the pathogenic *Fusarium* played a main role in the control treatment. Besides, the *Fusarium* population was very low in the fumigated treatment, so this might not be an issue. As a kind of biological control agent, the genus *Trichoderma* could compete nutrients and space with pathogens, alter soil conditions, promote the growth of plant, or some direct biocontrol such as mycoparasitism and antibiosis to exert biocontrol against fungal pathogen (Benítez et al. 2004). These beneficial species (*Bacillus* and *Trichoderma*) introduced by bio-fertilizer were detected at increased relative abundances in the FBOF treatment. Linear models revealed that higher relative abundance of *Bacillus*/*Trichoderma* might generate negative effect on *Fusarium* relative abundances. Therefore, the colonization of introduced *Bacillus* and *Trichoderma* after fumigation in our experiment had a direct effect on pathogen and, consequently, on the suppression of *Fusarium* wilt disease.

Several previous studies have illustrated that the biological organic fertilizer amendment played a major role in shaping soil microbial community composition (Ling et al. 2014; Qiu et al. 2012). Our results demonstrated that empty niches created due to fumigation are filled by microbial populations introduced by biological organic fertilizer which leads to interactions with the surviving resident populations. In this manner, the remodeling of the soil microbiome, in concert with potential antagonistic capacities, serve to suppress plant disease (Akhtar and Malik 2000).

Microbial diversity contains not only the abundance of species but also the complex interactions among different species (Olesen et al. 2007). Based on ecosystem theory, the sensibility of soil ecosystems to invader rest with their complexity (Fließbach et al. 2009). The use of phylogenetic molecular ecological networks (pMENs) is one of the methods to investigate the complexity of interactions within a microbial community. The fumigated (FOF and FBOF) treatments exhibited a lower number of nodes and links compared with OF treatment, due to the reduced soil microbial community complexity and stability after fumigation. However, compared to the FOF treatment, the FBOF treatment exhibited a higher number of nodes and links and a higher ratio of positive/negative links. Our results indicated that the re-shaped microbial community resulted in more microbial cooperation than competition.

Each soil microbial ecological network is made up of a series of nodes, and each node has a different role in the network topological structure. Although the number of module hubs in fumigation treatments was under non-fumigated treatment, the FBOF treatment contained more module hubs than FOF. This suggests that even though fumigation decreased the overall number of interactions, the biological organic fertilizer amendment resulted in a less drastic decrease in network complexity than organic fertilizer alone. Previous theory predicts that a group of species with higher probability of interacting with each other shows higher resistance and resilience against distribution owing to their buffering of the extinctions (Stouffer and Bascompte 2011). The majority of the module hubs in the FBOF treatment were bacteria, indicating that biological organic fertilizer amendment stimulated the microbial network to recover towards a bacterial- dominant community. Our previous research has demonstrated that *Fusarium* wilt disease may be more susceptible in fungal- dominant microbial communities (Zhao et al. 2017).

Only three module hubs were identified as fungi. Two of these were identified as the genus *Trichoderma* and the other one (OTU fun3928) was identified within the order *Hypocreaceae*. *Trichoderma* belong to the order *Hypocreaceae*, thus OTU fun3928 may have similar functions in *Fusarium* disease suppression as *Trichoderma*. However, additional research is necessary to support this hypothesis. Two module hubs within the FBOF treatment were assigned to *Bacillus*. Overall, our results indicate that the introduced species (*Bacillus* and *Trichoderma*) within the biological organic fertilizer which were significantly negatively correlated to *Fusarium* relative abundance, may be pivotal in the restoration of a suppressive soil microbial community.

**Fig. 5:**
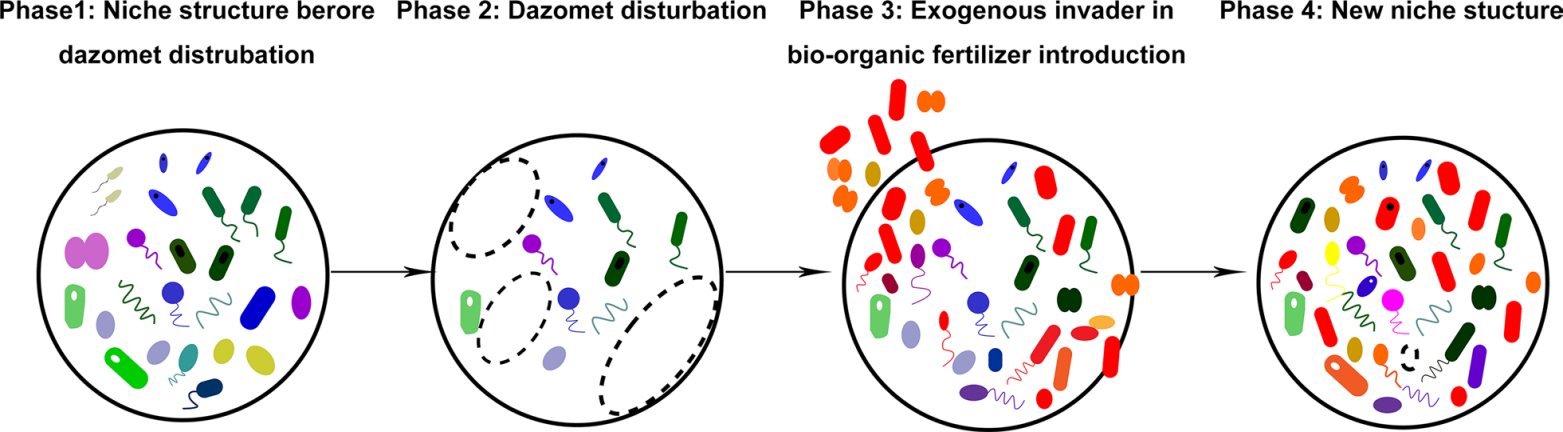
A conceptual model for understanding fumigation followed by biological organic fertilizer impacts on soil resident microbial community. Consider a microbial community of resident soil as a circle, as shown in (**a**). When dazomet is applied into the soil, the niches in resident soil are released (**b**). The introduced species in biological organic fertilizer will take up the niches (**c**) and alter the niche structure in such way (**d**)

A graphical and conceptual interpretation of the mechanism under the observed results is therefore hypothesized (Fig. 5). The initial disturbance by dazomet destroy the competition between numerous microbial taxa, which touches off a competitive release then exogenous species can more easily occupy the free niches. Consequently, the soil microbial community composition is re-arranged, and the niche differentiation pattern of the resident community is altered. This alteration in the community composition with biological organic fertilizer amendment then results in the establishment of a disease-suppressive community that acts through a combination of direct and indirect antagonism towards the pathogen.

**Fig. 6:**
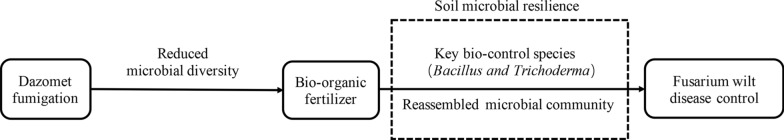
A conceptual cartoon summarizing the influence of dazomet followed by biological organic fertilizer treatments on soil microbial community and their influence on watermelon *Fusarium* wilt disease suppression

In conclusion, the result of this study shows that dazomet fumigation followed by biological organic fertilizer application effectively controlled watermelon *Fusarium* wilt disease, and is likely attributed to a combination of both the direct suppression of the pathogen population and the re-shaping of the soil microbiome. Fumigation disturbance resulted in a strong reduction in microbial community diversity. This appears to result in niche release and the drastic reduction in interactions within the soil microbial community. The beneficial species (*Bacillus* and *Trichoderma*), introduced through biological organic fertilizer amendment re-colonize the vacant niches and, through their interactions, manipulate the composition of the soil microbial community. This promotes a reversion to a relatively complex microbial community. Thus, the overall effectiveness of fumigation followed by biological organic fertilizer application appears to be a combination of introduced microbial direct effects on *Fusarium* and the promotion of changes in microbial community composition promoted by the biological organic fertilizer (Fig. 6). Therefore, we propose that manipulating the community re-assembly that occurs after fumigation is essential to promote sustainable plant health in the face of a growing worldwide pathogenic threat.

## Supplementary Information

**Additional file 1**: **Table S1. **Spearman’s correlations based on Bray-Curtis distance between Fusarium wilt disease incidence and microbial community composition determined by Mantel test. **Table S2.** Topological properties of the empirical phylogenetic molecular ecological networks (pMENs) among different treatments in comparison to the random networks. **Table S3.** Phylogenetic relationships of special OTUs in Zi-Pi plot of bulk and rhizosphere soil. **Figure S1. **Effect of different treatments on the population of pathogens. OF: Organic fertilizer, FOF: Dazomet fumigation coupled with organic fertilizer, FBOF: Dazomet fumigation coupled with bio-organic fertilizer. Different letters indicate significant differences among the treatments, as defined by Duncan test (*P* < 0.05). **Figure S2.** The relative abundance of bacterial phyla (A) and fungal genus (B) in the three treatments. OF: Organic fertilizer, FOF: Dazomet fumigation coupled with organic fertilizer, FBOF: Dazomet fumigation coupled with bio-organic fertilizer. **Figure S3.** Manhattan plots showing soil enriched OTUs in bacterial (A) and fungal (B) microbial communities between FBOF and OF treatment. OF: Organic fertilizer, FBOF: Dazomet fumigation coupled with bio-organic fertilizer. The dashed line corresponds to the false discovery rate-corrected P value threshold of significance (α = 0.05). The color of each dot represents the different taxonomic affiliation of the OTUs (phyla level), and the size corresponds to their RAs in the respective samples. **Figure S4.** Zi-Pi plot showing the distribution of OTUs based on their topological roles. OF: Organic fertilizer, FOF: Dazomet fumigation coupled with organic fertilizer, FBOF: Dazomet fumigation coupled with bio-organic fertilizer. Each symbol represents an OTU in network. The threshold values of Zi and Pi for categorizing OTUs were 2.5 and 0.62, respectively.

## Data Availability

All data generated or analyzed during this study are included in this published article (and its Additional file).
