# Supplementary material for: Manipulating the soil microbiomes during a community recovery process with plant beneficial species for the suppression of Fusarium wilt of watermelon
Source: AMB Express. 2021 Jun 12;11:87. doi: 10.1186/s13568-021-01225-5 (PMC8197695; doi:10.1186/s13568-021-01225-5)
Supplement: Supplementary file 1 — Additional file 1: Table S1. Spearman’s correlations based on Bray-Curtis distance between Fusarium wilt disease incidence and microbial community composition determined by Mantel test. Table S2. Topological properties of the empirical phylogenetic molecular ecological networks (pMENs) among different treatments in comparison to the random networks. Table S3. Phylogenetic relationships of special OTUs in Zi-Pi plot of bulk and rhizosphere soil. Figure S1. Effect of different treatments on the population of pathogens. OF: Organic fertilizer, FOF: Dazomet fumigation coupled with organic fertilizer, FBOF: Dazomet fumigation coupled with bio-organic fertilizer. Different letters indicate significant differences among the treatments, as defined by Duncan test (P < 0.05). Figure S2. The relative abundance of bacterial phyla (A) and fungal genus (B) in the three treatments. OF: Organic fertilizer, FOF: Dazomet fumigation coupled with organic fertilizer, FBOF: Dazomet fumigation coupled with bio-organic fertilizer. Figure S3. Manhattan plots showing soil enriched OTUs in bacterial (A) and fungal (B) microbial communities between FBOF and OF treatment. OF: Organic fertilizer, FBOF: Dazomet fumigation coupled with bio-organic fertilizer. The dashed line corresponds to the false discovery rate-corrected P value threshold of significance (α = 0.05). The color of each dot represents the different taxonomic affiliation of the OTUs (phyla level), and the size corresponds to their RAs in the respective samples. Figure S4. Zi-Pi plot showing the distribution of OTUs based on their topological roles. OF: Organic fertilizer, FOF: Dazomet fumigation coupled with organic fertilizer, FBOF: Dazomet fumigation coupled with bio-organic fertilizer. Each symbol represents an OTU in network. The threshold values of Zi and Pi for categorizing OTUs were 2.5 and 0.62, respectively. [file 13568_2021_1225_MOESM1_ESM.docx]

**Additional file 1**

**Table S1.** Spearman's correlations based on Bray-Curtis distance between Fusarium wilt disease incidence and microbial community composition determined by Mantel test

|  | rho | *P* |
| --- | --- | --- |
| Bacteria | 0.30 | 0.002 |
| Fungi | 0.52 | 0.001 |

The dissimilarity matrices of disease incidence, bacterial and fungal community composition were based on Bray-Curtis distance.

**Table S2.** Topological properties of the empirical phylogenetic molecular ecological networks (pMENs) among different treatments in comparison to the random networks

| Treatment | Community Empirical networks | | | | | | | | | Random netwoks | | |
| --- | --- | --- | --- | --- | --- | --- | --- | --- | --- | --- | --- | --- |
|  | Similarity  threshold  (*St*) | Total  links | Total  nodes | R^2^ of  power  law | Avg  connect  (avgK) | Harmonic  geodesic  distance  (HD) | Avg  clustering  coefficient  (avgCC) | Modularity  (No. of  modules) |  | Harmonic  geodesic  distance  (HD) | Avg  clustering  coefficient  (avgCC) | Modularity |
| OF | 0.92 | 1857 | 1118 | 0.92 | 3.322 | 7.829 | 0.157 | 0.859(78) |  | 4.338±0.028 | 0.011±0.003 | 0.593±0.003 |
| FOF | 0.92 | 286 | 339 | 0.95 | 1.687 | 2.572 | 0.100 | 0.958(78) |  | 6.222±0.754 | 0.004±0.003 | 0.910±0.008 |
| FBOF | 0.92 | 1241 | 603 | 0.84 | 4.116 | 3.366 | 0.199 | 0.706(67) |  | 3.503±0.030 | 0.034±0.004 | 0.479±0.004 |

OF: Organic fertilizer, FOF: Dazomet fumigation coupled with organic fertilizer, FBOF: Dazomet fumigation coupled with bio-organic fertilizer.

**Table S3.** Phylogenetic relationships of special OTUs in Zi-Pi plot of bulk and rhizosphere soil

| Treatment | ID | Generalists | Kingdom | Family | Genus |
| --- | --- | --- | --- | --- | --- |
| OF | bac96903 | Module hub | Bacteria | *Unclassified* | *Unclassified* |
|  | bac809485 | Module hub | Bacteria | *Cytophagaceae* | *Unclassified* |
|  | bac984280 | Module hub | Bacteria | *Nitrospiraceae* | *Nitrospira* |
|  | bac678259 | Module hub | Bacteria | *Unclassified* | *Unclassified* |
|  | bac249472 | Module hub | Bacteria | *A4b* | *Unclassified* |
|  | bac673969 | Module hub | Bacteria | *Unclassified* | *Unclassified* |
|  | bac576136 | Module hub | Bacteria | *Unclassified* | *Unclassified* |
|  | bac583206 | Module hub | Bacteria | *Unclassified* | *Unclassified* |
|  | bac50639 | Module hub | Bacteria | *Unclassified* | *Unclassified* |
|  | bac331521 | Module hub | Bacteria | *0319-6A21* | *Unclassified* |
|  | bac68830 | Module hub | Bacteria | *Methylophilaceae* | *Unclassified* |
|  | bac691349 | Module hub | Bacteria | *Sinobacteraceae* | *Unclassified* |
|  | bac87557 | Module hub | Bacteria | *0319-6A21* | *Unclassified* |
|  | bac128310 | Module hub | Bacteria | *SJA-101* | *Unclassified* |
|  | bac3435 | Module hub | Bacteria | *Unclassified* | *Unclassified* |
|  | bac252274 | Module hub | Bacteria | *Unclassified* | *Unclassified* |
|  | bac4430089 | Module hub | Bacteria | *Xanthomonadaceae* | *Unclassified* |
|  | bac860929 | Module hub | Bacteria | *Erythrobacteraceae* | *Unclassified* |
|  | bac1109964 | Module hub | Bacteria | *Sphingomonadaceae* | *Kaistobacter* |
|  | bac1084157 | Module hub | Bacteria | *Intrasporangiaceae* | *Unclassified* |
|  | bac187187 | Module hub | Bacteria | *Unclassified* | *Unclassified* |
|  | bac556903 | Module hub | Bacteria | *Nannocystaceae* | *Plesiocystis* |
|  | bac968336 | Module hub | Bacteria | *Alicyclobacillaceae* | *Alicyclobacillus* |
|  | bac512378 | Module hub | Bacteria | *Nocardioidaceae* | *Unclassified* |
|  | bac91318 | Module hub | Bacteria | *SJA-101* | *Unclassified* |
|  | bac1108483 | Module hub | Bacteria | *Unclassified* | *Unclassified* |
|  | bac3498923 | Module hub | Bacteria | *Unclassified* | *Unclassified* |
|  | bac120201 | Module hub | Bacteria | *Unclassified* | *Unclassified* |
|  | bac606989 | Module hub | Bacteria | *Xanthomonadaceae* | *Unclassified* |
|  | bac320782 | Module hub | Bacteria | *Sinobacteraceae* | *Steroidobacter* |
|  | bac827089 | Module hub | Bacteria | *Bacillaceae* | *Bacillus* |
|  | bac105606 | Module hub | Bacteria | *Micromonosporaceae* | *Pilimelia* |
|  | fun116136 | Module hub | Fungi | *Unclassified* | *Unclassified* |
|  | fun128596 | Module hub | Fungi | *Incertae sedis* | *Cephaliophora* |
|  | fun41686 | Module hub | Fungi | *Unclassified* | *Unclassified* |
|  | fun61187 | Module hub | Fungi | *Pleosporaceae* | *Alternaria* |
|  | fun127932 | Module hub | Fungi | *Unclassified* | *Unclassified* |
|  | fun70929 | Module hub | Fungi | *Unclassified* | *Unclassified* |
|  | fun127811 | Module hub | Fungi | *Unclassified* | *Unclassified* |
|  | fun68385 | Module hub | Fungi | *Hypocreaceae* | *Hypocrea* |
|  | fun128510 | Module hub | Fungi | *Incertae sedis* | *Acremonium* |
|  | fun50632 | Module hub | Fungi | *Hypocreaceae* | *Hypocrea* |
|  | fun17327 | Module hub | Fungi | *Nectriaceae* | *Fusarium* |
| FOF | bac336578 | Module hub | Bacteria | *A4b* | *Unclassified* |
|  | bac109242 | Module hub | Bacteria | *Sphingomonadaceae* | *Unclassified* |
|  | bac547097 | Module hub | Bacteria | *Unclassified* | *Unclassified* |
|  | bac215375 | Module hub | Bacteria | *Hyphomicrobiaceae* | *Unclassified* |
|  | bac778196 | Module hub | Bacteria | *Unclassified* | *Unclassified* |
|  | bac798097 | Module hub | Bacteria | *Hyphomicrobiaceae* | *Devosia* |
| FBOF | bac336578 | Module hub | Bacteria | *A4b* | *Unclassified* |
|  | bac238281 | Module hub | Bacteria | *Unclassified* | *Unclassified* |
|  | bac113486 | Module hub | Bacteria | *Unclassified* | *Unclassified* |
|  | bac552687 | Module hub | Bacteria | *Sphingomonadaceae* | *Unclassified* |
|  | bac13733 | Module hub | Bacteria | *Unclassified* | *Unclassified* |
|  | bac919491 | Module hub | Bacteria | *Bacillaceae* | *Bacillus* |
|  | bac2885855 | Module hub | Bacteria | *Bacillaceae* | *Bacillus* |
|  | bac111733 | Module hub | Bacteria | *Unclassified* | *Unclassified* |
|  | bac113224 | Module hub | Bacteria | *Unclassified* | *Unclassified* |
|  | bac591647 | Module hub | Bacteria | *Unclassified* | *Unclassified* |
|  | fun58209 | Module hub | Fungi | *Hypocreaceae* | *Trichoderma* |
|  | bac128235 | Module hub | Bacteria | *Unclassified* | *Unclassified* |
|  | bac83047 | Module hub | Bacteria | *Unclassified* | *Unclassified* |
|  | bac77336 | Module hub | Bacteria | *Comamonadaceae* | *Ramlibacter* |
|  | bac722895 | Module hub | Bacteria | *Sphingomonadaceae* | *Sphingopyxis* |
|  | fun3928 | Module hub | Fungi | *Hypocreaceae* | *unidentified* |
|  | fun11782 | Module hub | Fungi | *Hypocreaceae* | *Trichoderma* |
|  | bac141276 | Module hub | Bacteria | *Piscirickettsiaceae* | *Unclassified* |
|  | bac767888 | Module hub | Bacteria | *Unclassified* | *Unclassified* |
|  | bac250298 | Module hub | Bacteria | *Micromonosporaceae* | *Catellatospora* |
|  | bac163965 | Module hub | Bacteria | *Unclassified* | *Unclassified* |
|  | bac519510 | Module hub | Bacteria | *Erythrobacteraceae* | *Unclassified* |
|  | bac112996 | Module hub | Bacteria | *Unclassified* | *Unclassified* |
|  | bac510591 | Connector | Bacteria | *Chitinophagaceae* | *Unclassified* |

OF: Organic fertilizer, FOF: Dazomet fumigation coupled with organic fertilizer, FBOF: Dazomet fumigation coupled with bio-organic fertilizer.

**Figure legend**

**
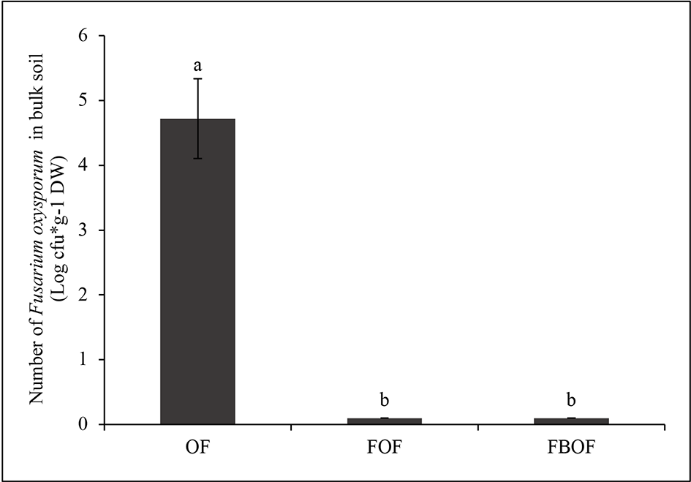
Figure S1.** Effect of different treatments on the population of pathogens. OF: Organic fertilizer, FOF: Dazomet fumigation coupled with organic fertilizer, FBOF: Dazomet fumigation coupled with bio-organic fertilizer. Different letters indicate significant differences among the treatments, as defined by Duncan test (*P* < 0.05).

**Figure S2.** The relative abundance of bacterial phyla (A) and fungal genus (B) in the three treatments.


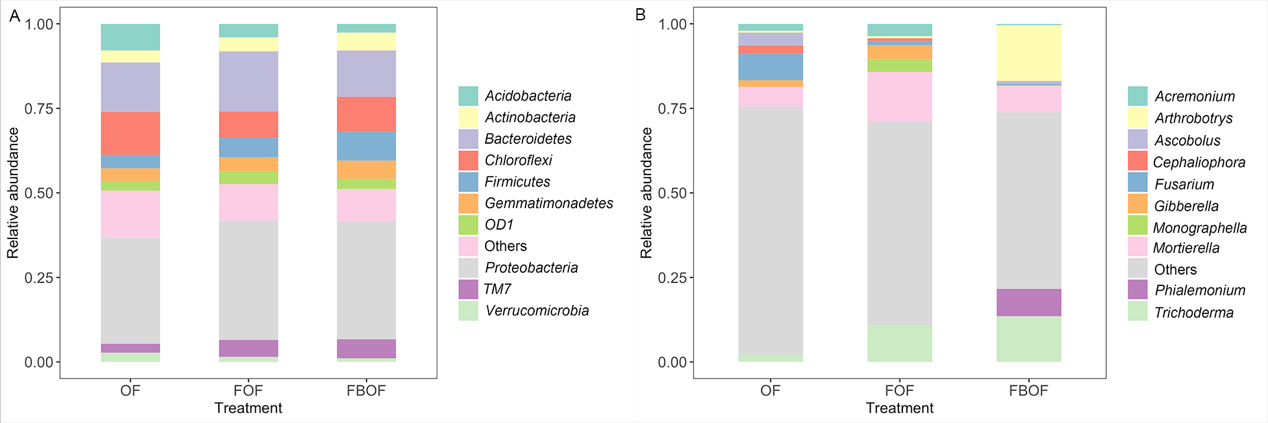


OF: Organic fertilizer, FOF: Dazomet fumigation coupled with organic fertilizer, FBOF: Dazomet fumigation coupled with bio-organic fertilizer.

**Figure S3.** Manhattan plots showing soil enriched OTUs in bacterial (A) and fungal (B) microbial communities between FBOF and OF treatment.


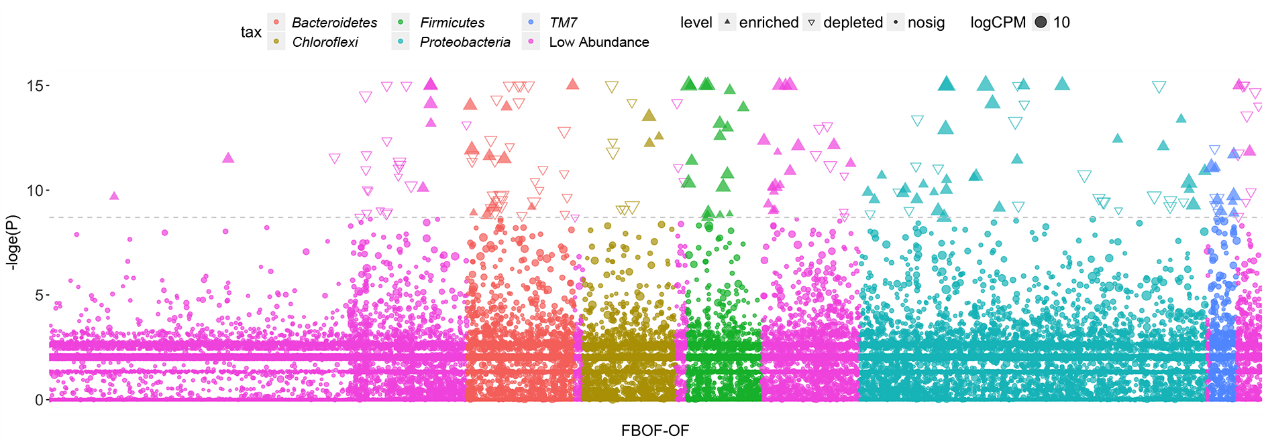


OF: Organic fertilizer, FBOF: Dazomet fumigation coupled with bio-organic fertilizer. The dashed line corresponds to the false discovery rate-corrected P value threshold of significance (α = 0.05). The color of each dot represents the different taxonomic affiliation of the OTUs (phyla level), and the size corresponds to their RAs in the respective samples.

**Figure S4.** Zi-Pi plot showing the distribution of OTUs based on their topological roles.


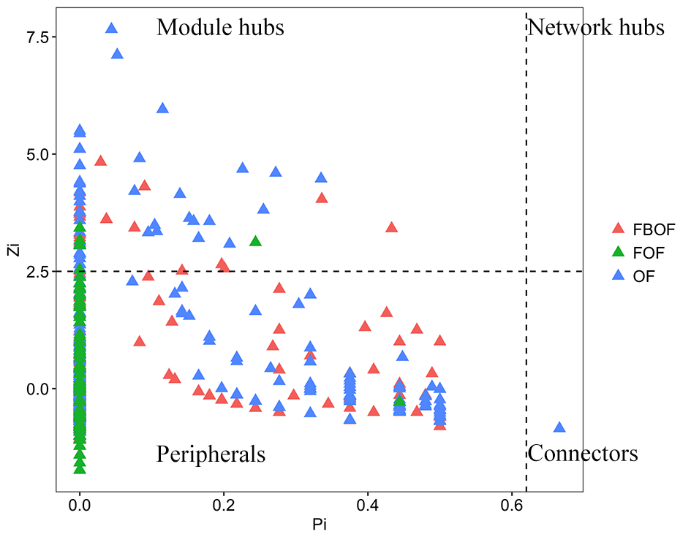


OF: Organic fertilizer, FOF: Dazomet fumigation coupled with organic fertilizer, FBOF: Dazomet fumigation coupled with bio-organic fertilizer. Each symbol represents an OTU in network. The threshold values of Zi and Pi for categorizing OTUs were 2.5 and 0.62, respectively.
